# Predictors of Physical Activity Among Older Breast Cancer Survivors: Findings From the WHI LILAC Study

**DOI:** 10.1093/geroni/igab046.1156

**Published:** 2021-12-17

**Authors:** Michael Pennell, Michelle Naughton, Xiaochen Zhang, Aladdin Shadyab, Candyce Kroenke, Nazmus Saquib, Electra Paskett, Jessica Krok-Schoen

**Affiliations:** 1 College of Public Health, The Ohio State University, Columbus, Ohio, United States; 2 Comprehensive Cancer Center, Columbus, Ohio, United States; 3 University of california, San Diego, San Diego, California, United States; 4 Kaiser Permanente Northern California Division of Research, Oakland, California, United States; 5 College of Medicine, Sulaiman Al Rajhi University, Bukairyah, Al Qasim, Saudi Arabia; 6 Department of Internal Medicine, College of Medicine, The ohio State University, Columbus, Ohio, United States; 7 The Ohio State University, Columbus, Ohio, United States

## Abstract

We examined the factors associated with physical activity following cancer treatment among older breast cancer survivors from the WHI LILAC study. The majority of participants (n=3,710, mean age=78.8±5.9) were white (86%), and had in situ/localized breast cancer (79%). Women who received radiation therapy, were underweight/normal weight, had fewer reported cancer-related symptoms, no lymphedema, higher self-rated health, higher physical functioning, no pain, no depressive symptoms, and higher social support had significantly greater participation and duration of physical activity (all p<0.05). Women aged <75 who received radiation had longer duration of total minutes of physical activity (β=19.7, p<0.05), while women aged 75-85 who received radiation had shorter duration of total minutes of physical activity (β=-3.2, p<0.05). These results indicate that multiple health and social factors are associated with physical activity in this cohort. Interventions to facilitate physical activity among this group should consider body weight, symptom burden, comorbidity status, and social support.

